# Artemisinin-derived dimer ART-838 potently inhibited human acute leukemias, persisted *in vivo*, and synergized with antileukemic drugs

**DOI:** 10.18632/oncotarget.6896

**Published:** 2016-01-12

**Authors:** Jennifer M. Fox, James R. Moynihan, Bryan T. Mott, Jennifer R. Mazzone, Nicole M. Anders, Patrick A. Brown, Michelle A. Rudek, Jun O. Liu, Ravit Arav-Boger, Gary H. Posner, Curt I. Civin, Xiaochun Chen

**Affiliations:** ^1^ Center for Stem Cell Biology & Regenerative Medicine, Departments of Pediatrics and Physiology, University of Maryland School of Medicine, Baltimore, MD 21201, USA; ^2^ Department of Chemistry, School of Arts and Sciences, Johns Hopkins University, Baltimore, MD 21218, USA; ^3^ Sidney Kimmel Comprehensive Cancer Center, Johns Hopkins University School of Medicine, Baltimore, MD 21231, USA; ^4^ Department of Pharmacology and Molecular Science, Johns Hopkins University School of Medicine, Baltimore, MD 21205, USA; ^5^ Department of Pediatrics, Johns Hopkins University School of Medicine, Baltimore, MD, 21231, USA; ^6^ Johns Hopkins Malaria Research Institute, Bloomberg School of Public Health, Baltimore, MD 21205, USA

**Keywords:** artemisinins, ART-838, leukemia, ROS, chemotherapy

## Abstract

Artemisinins, endoperoxide-containing molecules, best known as antimalarials, have potent antineoplastic activity. The established antimalarial, artesunate (AS), and the novel artemisinin-derived trioxane diphenylphosphate dimer 838 (ART-838) inhibited growth of all 23 tested acute leukemia cell lines, reduced cell proliferation and clonogenicity, induced apoptosis, and increased intracellular levels of reactive oxygen species (ROS). ART-838 was 88-fold more potent that AS in vitro, inhibiting all leukemia cell lines at submicromolar concentrations. Both ART-838 and AS cooperated with several established antileukemic drugs and newer kinase inhibitors to inhibit leukemia cell growth. ART-838 had a longer plasma half-life than AS in immunodeficient NOD-SCID-IL2Rg^null^ (NSG) mice, remaining at effective antileukemic concentrations for >8h. Intermittent cycles of ART-838 inhibited growth of acute leukemia xenografts and primagrafts in NSG mice, at higher potency than AS. Based on these preclinical data, we propose that AS, with its established low toxicity and low cost, and ART-838, with its higher potency and longer persistence *in vivo*, should be further developed toward integration into antileukemic regimens.

## INTRODUCTION

Artemisinin, a natural antimalarial compound, is the active component of the sweet wormwood plant (*Artemisia annua* L.) used in traditional Chinese medicine to treat fevers [1]. Numerous artemisinin analogs with improved pharmacological properties have been developed, and artesunate (AS) is the World Health Organization-recommended treatment for severe malaria. Artemisinins also inhibit growth of a broad range of microbes and cancer cells, especially leukemias [2–4]. Artemisinins appear to inhibit cancers by mechanisms that differ from those of established antineoplastic agents, and chemotherapy-resistant leukemia and neuroblastoma cell lines remain sensitive to artemisinins [2, 5, 6]. The accepted basis for both the antimicrobial and the antineoplastic activity of artemisinins is bioactivation of the endoperoxide pharmacophore(s) by heme iron to carbon-centered radicals, resulting in ROS generation via the electron transport chain and subsequent apoptosis [6–15].

Although first-generation artemisinin derivatives such as AS have enhanced antimalarial and antineoplastic activity compared to natural artemisinin, they are rapidly catabolized *in vivo* to the active metabolite dihydroartemisinin (DHA), which is then glucuronidated and excreted [16]. The novel semi-synthetic artemisinin-derived trioxane diphenylphosphate dimer 838 (ART-838) exhibits greater antineoplastic and antiviral activity than monomeric AS [8, 11, 17–21]. In our recent structure-activity relationship study of artemisinin trioxane dimers, we identified ART-838 as an exquisitely potent antileukemic drug, with a nearly 70-fold lower IC_50_ than that of AS against Jurkat T-cell acute lymphoblastic leukemia (T-ALL) cells [22]. In addition, we demonstrated a favorable therapeutic window for ART-838, wherein it inhibited growth of leukemia cells but not normal peripheral blood mononuclear cells, similar to results of ART-838 in solid tumor cell lines compared to normal fibroblasts [11].

In this study, we demonstrated in vitro efficacy of AS and ART-838 against 23 human acute leukemia cell lines and involvement of iron-dependent ROS generation in these anti-proliferative and pro-apoptotic effects. ART-838 had superior pharmacokinetics (PK) following oral administration to mice than AS. Treatment with AS or ART-838 inhibited *in vivo* growth of human AML xenografts and B-precursor ALL (B-ALL) primagrafts, and AS and ART-838 potentiated the in vitro anti-proliferative effects of 6 established or emerging antileukemic drugs. Since AS is inexpensive and established as safe in humans through extensive use against malaria, it is a promising current candidate to be repurposed for acute leukemia treatment. Although further preclinical and clinical testing will be required for ART-838, this new compound offering higher potency and extended *in vivo* half-life might replace AS in the future.

## RESULTS

### ART-838, like AS, potently inhibited acute leukemia growth and clonogenicity

**Figure 1:**
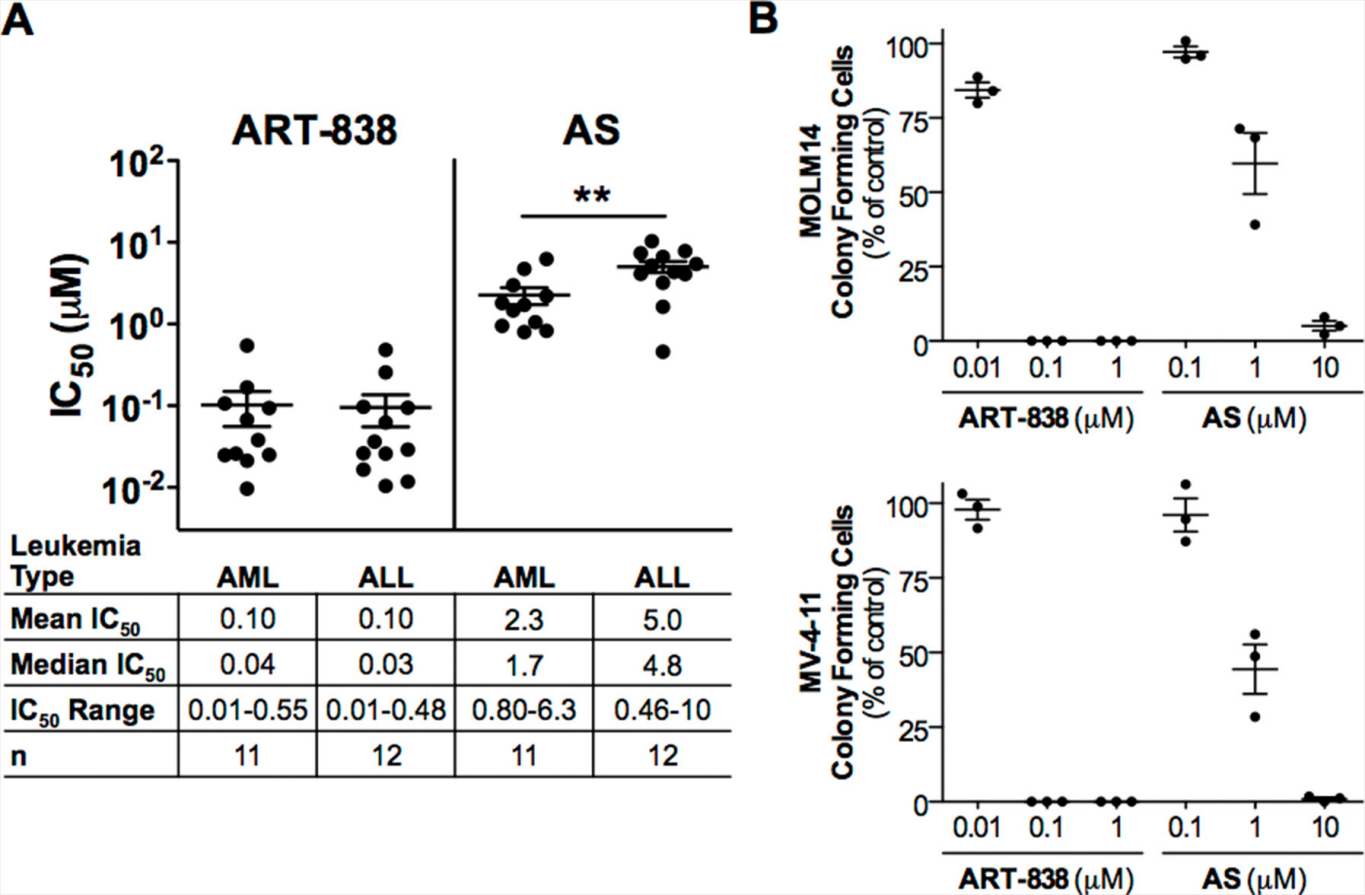
ART-838 inhibited human acute leukemia cell growth and colony formation, more potently than did AS **A.** 23 human leukemia cell lines were treated with increasing concentrations of ART-838 or AS for 48h, and then cytotoxicity was assessed via fluorescent alamarBlue assay. Following subtraction of background fluorescence from all wells, fluorescence of drug-treated cells was normalized to that of vehicle (<0.5% DMSO)-treated cells to determine the effect of each drug concentration on viability. Each cell line was tested in triplicate and in at least three independent experiments. IC_50_ (concentration that inhibits cell growth by 50%) values were calculated using CompuSyn software (ComboSyn, Paramus, NJ). All IC_50_s were determined using cells cultured in RPMI containing 10% FBS. In addition several cell lines were initially cultured and tested in alternative media (Supplementary Table S2). Bisecting lines indicate mean IC_50_s and error bars, SEM (**, *p*<.01) **B.** MOLM14 (AML) or MV-4-11 (biphenotypic leukemia) cells were pretreated with the indicated concentrations of ART-838, AS, or vehicle (0.5% DMSO) for 24h then cultured in semi-solid medium, in triplicate, for 7-10d before colonies were enumerated. Colony counts were normalized to vehicle-treated controls (100%). Three independent experiments were performed.

ART-838 inhibited growth of all 23 leukemia cell lines tested (IC_50_ range: 0.01-0.55 μM; Figure 1A, Supplementary Table S1). ART-838 was 11-315-fold (average 88-fold) more potent than AS (IC_50_ range: 0.46-10.3 μM), but ART-838 and AS IC_50_s significantly correlated (Supplementary Figure S1, p<.01). Overall, AML and ALL cell lines were equally sensitive to ART-838, while AMLs were slightly more sensitive to AS than were ALLs (Figure 1A). Cell lines harboring mixed lineage leukemia gene rearrangements (MLLr) were slightly but not significantly more sensitive to both ART-838 and AS than cell lines without MLLr (Supplementary Figure S2). The presence of p53 mutations did not correlate with drug sensitivity to either ART-838 or AS (Supplementary Table S1). Time-dependent growth inhibition of the moderately sensitive SEM and THP-1 cell lines was evident over 96h at 0.1 μM and 1 μM ART-838, as well as 1 μM and 10 μM AS (Supplementary Figure S3). At the same concentrations, near complete growth inhibition was observed within 24-48h in the highly sensitive KOPN8 and MOLM14 cell lines.

Similar to the above results from bulk population assays, ART-838 was considerably more potent than AS in reducing leukemia clonogenicity. ART-838 (0.1 μM) completely abolished, and AS (10 μM) substantially reduced, clonogenicity of MOLM14 and MV-4-11 cells (Figure 1B); ART-838 and AS also reduced clonogenicity of K562 and HL-60 cells (Supplementary Figure S4). In contrast, treatment with 1 μM ART-838 or 10 μM AS reduced normal erythroid and monogranulocytic colony-forming cells by <25% (Supplementary Figure S5A, S5B).

### ART-838, like AS, inhibited cell cycle progression and induced caspase-dependent apoptosis

**Figure 2:**
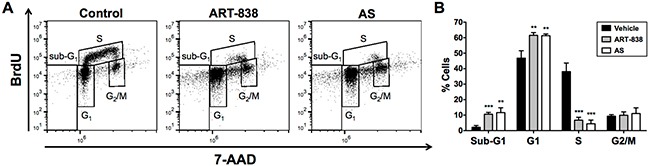
ART-838 inhibited human acute leukemia cell cycle progression more potently than did AS MOLM14 cells treated with ART-838 (0.1 μM) or AS (5 mM) for 24h were analyzed for cell cycle distribution by measuring DNA synthesis (BrdU incorporation) and DNA content (7-AAD) via flow cytometry, using the FL3 and FL4 channels of an Accuri C6 flow cytometer (BD Biosciences). After cell debris was gated out using FSC/SSC parameters, 10,000 cellular events were collected per sample. Data was analyzed and fluorescence compensation performed using FlowJo software, version 10 (Tree Star, Ashland, OR). **A.** Representative plots from a single experiment. **B.** Quantification of the percentage cells in each phase of the cell cycle from three independent flow cytometry experiments (**, *p*<.01 vs control; ***, *p*<.001 vs control).

Cell cycling was assessed in MOLM14 AML cells treated with 0.1 μM ART-838 or 5 μM AS for 24h. These concentrations caused 90-99% growth inhibition by 48h in alamarBlue assays (Supplementary Figure S6). By 24h, both ART-838 and AS caused significant reductions in S-phase cells (38% in vehicle-treated cells vs <7% in ART-838- or AS-treated cells; Figure 2) accompanied by significant increases in sub-G_1_-phase (2% vs >10%) and G_1_-phase (47% vs >61%) cells.

### ART-838, like AS, induced caspase-dependent apoptosis

**Figure 3:**
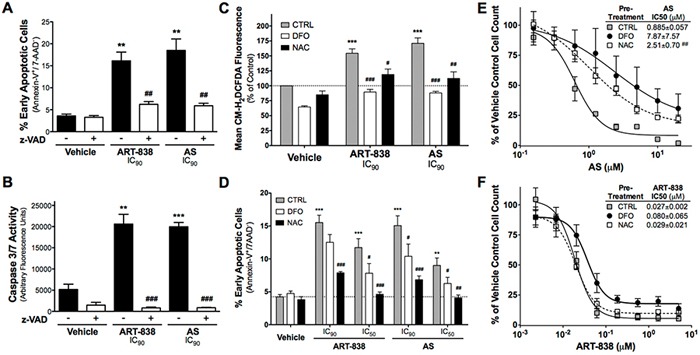
ART-838 induced Caspase-mediated apoptosis and ROS generation in MOLM14 cells more potently than did AS **A-B.** Cells were pretreated with z-VAD (50 μM) for 1h, and then incubated with ART-838 (0.1 μM) or AS (5 μM) for 24h. **A.** Quantification of the percentage of early apoptotic cells (Annexin-V+/7-AAD-) from three independent experiments in which cells were stained for apoptosis with 7-AAD and Annexin-V then analyzed by flow cytometry. (B) Cells were analyzed for Caspase-3/7 activity using a fluorescent peptide substrate after z-VAD pretreatment and 24h ART-838 or AS treatment. **C.** Cells were pre-loaded with CM-H_2_DCFDA (5 mM) for 30min, pretreated with DFO (11 mM) or NAC (25 μM) for 60-120min, and then treated with ART-838 (IC_90_: 0.1 μM) or AS (IC_90_: 5 μM). Preliminary experiments showed that DFO and NAC were cytotoxic to MOLM14 cells at high concentrations, so each was used at its approximate 48h IC_50_ (concentration that inhibited growth by only 50% at 48h). 16h after addition of drugs, cells were washed and analyzed by flow cytometry for CM-H_2_DCFDA fluorescence (indicating total ROS generation) in the FL1 channel. Mean Fluorescence Intensity (MFI) was normalized to that of vehicle control (0.5% DMSO) with no pretreatment. **D.** Cells were pretreated with DFO or NAC as in C, then treated with ART-838 (IC_50_: 0.025 μM, IC_90_: 0.1 μM) or AS (IC_50_: 0.825 μM, IC_90_: 5 μM) for 24h before analyzing for apoptosis as in A. **E, F.** Cells were pretreated with DFO or NAC as in C, then incubated with a range of concentrations of AS (E) or ART-838 (F). for 48h. Viable cell counts, obtained via Trypan Blue dye exclusion, were normalized to vehicle (0.02% DMSO)-treated samples +/– pretreatment. [**, *p*<.01 and ***, *p*<.001 for ART-838 or AS vs control (far left bars); #, *p*<.05, ##, *p*<.01, and ###, *p*<.001 for ART-838 or AS with vs without inhibitor pretreatment].

AS (5 μM) induced significant apoptosis and caspase-3/7 activation in MOLM14 cells (Figure 3A, 3B), as previously demonstrated in T-ALL cell lines [7]. Similar levels of apoptosis and caspase-3/7 activity were achieved with a 50-fold lower ART-838 concentration. Pretreatment with the pan-caspase inhibitor z-VAD(OMe)-fmk (z-VAD) inhibited caspase-3/7 activity and apoptosis in ART-838-treated and AS-treated cells. ART-838 and AS similarly inhibited cell cycle progression and induced apoptosis of KOPN8 B-ALL cells (not shown).

### Iron and ROS contributed to the antileukemic activity of ART-838 and AS

Consistent with the importance of the endoperoxides for ART-838's antileukemic activity, IC_50_s for an ART-838 derivative lacking both endoperoxides were >1000-fold higher than those for ART-838 against both KOPN8 and MOLM14 cells (deoxy-ART-838, Supplementary Table S3). In addition, both ART-838 (0.1 μM) and AS (5 μM) significantly increased total cellular ROS levels in MOLM14 cells (Figure 3C).

Pretreatment with the iron chelator deferoxamine mesylate (DFO; 11 μM) ablated cellular ROS generation and reduced apoptosis by AS and ART-838 (Figure 3C, 3D). The ROS scavenger N-acetyl cysteine (NAC; 25 μM) significantly reduced ROS generation and apoptosis by ART-838 and AS (Figure 3C, 3D). MOLM14 cells pretreated with DFO or NAC gained 9- or 3-fold resistance to growth inhibition by AS respectively (Figure 3E). The same pretreatment with DFO induced 3-fold resistance to ART-838, but resistance to ART-838 was not increased by the NAC pretreatment (Figure 3F). Since pretreatment with >25 μM NAC, alone, caused significant inhibition of MOLM14 cell growth, we could not examine the effect of higher NAC concentrations on sensitivity to the more potent ART-838. Nevertheless, the lack of inhibition of ART-838-mediate antileukemic efficacy by this standard dose of NAC suggests that mechanisms beyond ROS might be important in the antileukemic activity of ART-838.

### PK of ART-838 in NSG mice

**Figure 4:**
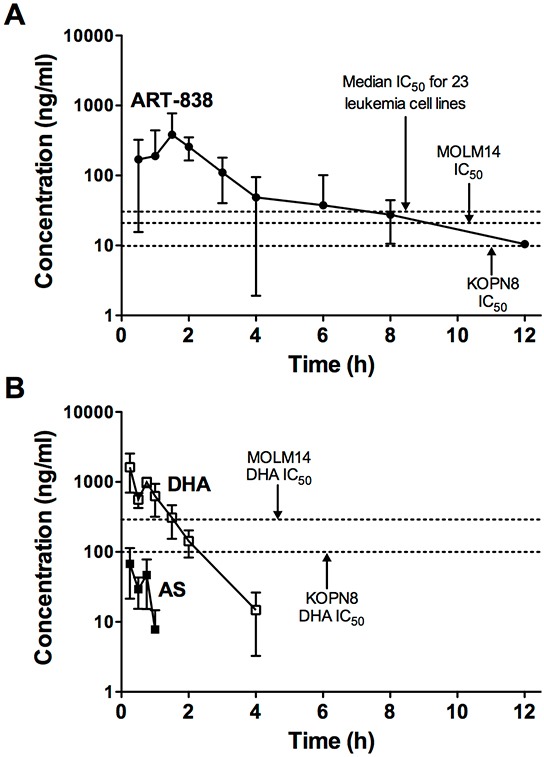
PKs of ART-838 and AS in NSG mice **A.** ART-838, **B.** AS, and DHA plasma concentrations in NSG mice treated with a single oral (gavage) dose of ART-838 (50 mg/kg) or AS (200 mg/kg). Plasma samples were obtained from 3-6 male NSG mice per time point (15min-24h after drug administration). Symbols represent concentrations of compound by LC/MS-MS detection. Dotted lines represent in vitro IC_50_s of DHA (Supplementary Table S3) or ART-838 (Supplementary Table S1) for MOLM14 and KOPN8 cells or the median ART-838 IC_50_ for all 23 tested leukemia cell lines (Supplementary Table S1). Detailed PK parameters are shown in Supplementary Table S4.

The maximum plasma concentration (C_max_) of ART-838 (383 ng/ml; 0.45 μM) occurred 1.5h after administration of a single 50 mg/kg oral dose to NSG mice (Supplementary Table S4). ART-838's plasma half-life (T_1/2_) was >3h, with plasma concentrations remaining above the MOLM14 in vitro IC_50_ for >8h (Figure 4A). ART-838's *in vivo* metabolites are unknown; DHA was one theoretical metabolite, as was the parent compound, ART-606 (a dimeric artemisinin linked by one carbon that would result if the diphenyl phosphate ester group were lost *in vivo*) [11]. However, we did not detect DHA or ART-606 after ART-838 administration. The total ART-838 exposure based on the area under the concentration-time curve (AUC) from time zero to the last concentration above the detection limit (AUC_last_) was 903 h*ng/ml. In contrast, AS was rapidly metabolized to DHA [23, 24], which had a T_1/2_ <1h, and AS and DHA declined to undetectable levels by 1h and 4h, respectively (Figure 4B). C_max_ for AS (67 ng/ml; 0.24 μM) and DHA (1629 ng/ml; 5.74 μM) occurred 0.25h after oral administration (Supplementary Table S4). DHA levels remained above the MOLM14 in vitro IC_50_ for ∼2h.

### ART-838 or AS treatment slowed subcutaneous MOLM14 xenograft growth

**Figure 5:**
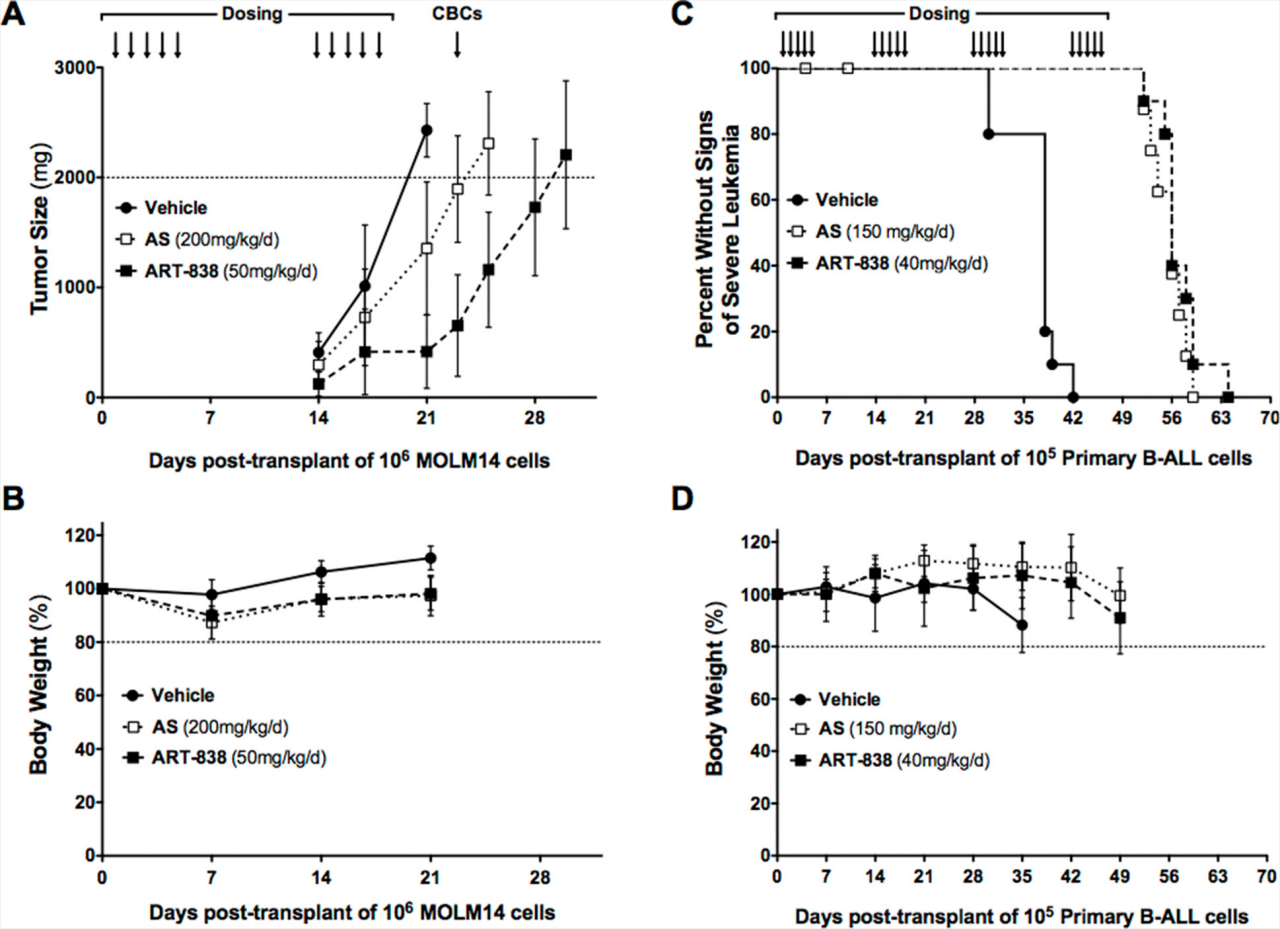
ART-838 inhibited growth of acute leukemia xenografts and primagrafts in NSG mice more potently than did AS **A, B.** NSG mice transplanted subcutaneously with MOLM14 AML cells (10^6^/mouse), were treated by oral gavage with two 5-day cycles of ART-838 (50 mg/kg/d, *n* = 8), AS (200 mg/kg/d, *n* = 9), or vehicle (n=10) beginning on days 1 and 14 post-transplantation (arrows indicate days of drug administration). Mice were weighed weekly and tumors measured at least twice weekly. CBCs were performed on day 23 (Supplementary Figure S8). Mice were euthanized when tumors reached an estimated 2000mg. Ascites was not observed in any mouse. **A.** Average tumor mass (estimated by: [length x width^2^]/2); (*p*<.001 for ART-838 or AS vs vehicle tumor mass at day 21). **B.** Percent of Day 0 body weight. (C,D) NSG mice (n=10 per group) transplanted with primary B-ALL case #109 (10^5^ cells/mouse), were treated by oral gavage with four 5-day cycles of ART-838 (40 mg/kg/d), AS (150 mg/kg/d), or vehicle beginning on days 1, 15, 29, and 43 post-transplantation (arrows indicate days of drug administration). Mice were weighed weekly and observed daily for clinical signs of severe leukemia. **C.** Kaplan-Meier survival analysis. Mean time to severe clinical signs of leukemia (surrogate for survival): vehicle (37), AS (56), and ART-838 (57); (*p*<.001 for ART-838 vs vehicle or AS vs vehicle). 2 mice from the AS group that died on days 4 and 10 were censored from analysis, as they showed no evidence of leukemia or drug toxicity at necropsy. **D.** Percent of Day 0 body weight.

NSG mice were transplanted with MOLM14 cells subcutaneously (sc) (day 0) and, beginning on days 1 and 14 post-transplantation, treated by oral gavage with two 5-day cycles of ART-838 (50 mg/kg/d, *n* = 8 mice), AS (200 mg/kg/d, *n* = 9 mice), or vehicle (control, *n* = 10 mice) (Figure 5A). In prior toxicity experiments, these had been determined to be approximate chronic maximum-tolerated doses (MTD) for this schedule. The dose-limiting toxicity was >20% weight loss, and no clinical neurotoxicity was observed [25]. Normal NSG mice treated with a 5-day cycle of AS or ART-838 at the above doses had normal complete and differential blood cell counts on days 6 and 12 (Supplementary Figure S7). In the treatment experiment shown in Figure 5A, mouse body weights recovered within 1 week after each 5-day treatment cycle (Figure 5B). Most mice developed palpable tumors by day 14, but in three ART-838-treated mice, tumors were not palpable until day 17. Vehicle-treated mice required euthanasia for 2000 mg tumors at day 21, at which time the average mass of ART-838-treated tumors was 83% less, and that of AS-treated tumors 44% less, than the average mass of vehicle-treated tumors (Figure 5A). Complete and differential blood cell counts were within the normal range on day 23, 5 days after the completion of the second treatment cycle (Supplementary Figure S8).

### ART-838 or AS treatment delayed onset of clinical leukemia in an intravenous B-ALL primagraft model

NSG mice transplanted with primary B-ALL case #109 cells intravenously (iv) on day 0 were treated by gavage with four 5-day cycles of ART-838 (40 mg/kg/d) or AS (150 mg/kg/d), beginning on days 1, 15, 29, and 43 post-transplantation (Figure 5C). No dose-limiting body weight loss following treatment was observed (Figure 5D). Although all mice eventually developed severe leukemia, with massive splenomegaly, pale bone marrow, and replacement of hematopoietic organs with human B-ALL cells, ART-838 or AS treatment significantly delayed leukemia onset (Figure 5C). ART-838 treatment extended mean survival time by 20 days (55% prolongation), and the 4-fold higher dose AS regimen extended mean survival time by 19 days (51% prolongation). In a second experiment, a slightly different treatment schedule (two 5-day cycles of ART-838 [50 mg/kg/d] or AS [200 mg/kg/d] beginning on days 1 and 29 post-transplantation) delayed leukemia to a similar extent (Supplementary Figure S9).

### ART-838 or AS enhanced the in vitro cytotoxicity of established and emerging antileukemic agents

MOLM14 or KOPN8 cells were treated with each of three standard antileukemic drugs (cytosine arabinoside [ARA], doxorubicin [DOX], etoposide [ETO]) or three kinase inhibitors (midostaurin [MID], lestaurtinib [LES], sorafenib [SOR]); each of which target multiple kinases, including FMS-like tyrosine kinase receptor (FLT3) [26], alone and in combination with ART-838 or AS at doses centered on an equipotency ratio based on the IC_50_s for each drug, as recommended for drug combination studies (Supplementary Table S3) [27]. Addition of ART-838 or AS enhanced the cytotoxicity of all six tested drugs across a broad range of concentrations (Supplementary Figures S10 and S11). Based on combination indices (CI) < 1 indicating synergy and CI = 1 indicating additivity, drug interactions were typically most favorable at higher drug concentrations.

## DISCUSSION

Both the novel artemisinin dimer ART-838 and the clinical antimalarial AS inhibited growth of all 23 acute leukemia cell lines tested. Consistent with multiple reports that dimeric artemisinin analogs are more cytotoxic toward cancer cells than their monomeric counterparts [7, 11, 20, 22], ART-838 was far more potent against MOLM14 and other leukemia cell lines than AS in assays of cell growth, cell cycling, and apoptosis. Indeed, ART-838 was effective with IC_50_<1μM against every acute leukemia tested, including those harboring poor prognosis mutations such as MLLr and FLT3 internal tandem duplication (FLT3/ITD) [26, 28].

Iron-activation of the endoperoxide pharmacophore, resulting in ROS generation, is critical to both the antimalarial and antineoplastic activities of artemisinins [6–15]. As previously demonstrated for AS in breast cancer cells [10], an iron-activation mechanism is supported by the reported enhanced antineoplastic activity of AS with the addition of ferrous iron [29–31]. ROS generation by ART-838 or AS was completely abolished by iron chelation with DFO, and both ART-838- and AS-induced apoptosis were partially inhibited by DFO pretreatment. Absence of the endoperoxide group from ART-838 abrogated its antileukemic activity. Our observation that NAC reduced the antileukemic efficacy of AS but not ART-838 suggests that ART-838 may trigger additional antileukemic mechanisms beyond ROS induction.

To begin optimizing treatment regimens for future clinical studies, we assessed the PK, tolerability, and efficacy of ART-838 and AS *in vivo*. The single dose MTDs for AS and ART-838 via oral gavage administration in NSG mice were ∼500-600 mg/kg, and the chronic MTDs (5 days/week by oral gavage) for ART-838 and AS were 40-50 mg/kg and 150-200 mg/kg respectively. In PK studies, AS was rapidly hydrolyzed to its active metabolite DHA, declining to undetectable levels by 1h, consistent with its known short T_1/2_ in humans [23, 24]. ART-838 was also well absorbed, but was not metabolized to DHA or ART-606, and had a ∼6-fold longer T_1/2_ than DHA, with concentrations remaining above the median IC_50_ for all 23 cell lines for >6h. The superior PKs of ART-838 vs AS/DHA may facilitate effective implementation into antileukemic regimens in the future.

Mice tolerated two 5d cycles of ART-838 (50 mg/kg/d) or AS (200 mg/kg/d). For both ART-838 and AS, the dose-limiting toxicity in NSG mice was acute weight loss, which has not been described in humans receiving AS [32, 33]. We found that the mice regained weight within a few days after the last ART-838 or AS dose. Mouse behavior was normal, and major organs appeared normal at postmortem. Mouse blood cell counts were not reduced, in contrast to human clinical results, where AS dose was limited by transient neutropenia [32]. For this reason and because transient neutropenia may be acceptable in leukemia patients, the possibility that humans may tolerate dose-intensive artemisinin administration should be carefully examined in Phase I/II studies in leukemia. Alternative routes of ART-838 and AS administration, e.g. intermittent or continuous infusion parenteral, could also be evaluated in preclinical models and/or clinical trials with accompanying PK and pharmacodynamic (PD) studies.

We used an sc xenograft tumor-formation model to begin testing *in vivo* efficacy of ART-838 and AS. The MOLM14 cell line was selected because of its sensitivity to ART-838 and AS in vitro, despite its clinically unfavorable MLLr and FLT3/ITD mutations. Both artemisinins significantly delayed sc tumor growth, and ART-838 was more effective than AS at an approximately equitoxic dose. To more rigorously assess the *in vivo* efficacy of ART-838 and AS, we used an iv primagraft model that more faithfully recapitulates primary leukemia growth in humans. Primary B-ALL case #109, harboring an MLLr mutation, was selected as the first ALL to test *in vivo*. Two different treatment regimens were tested in this iv primagraft model: 1) two 5d cycles of ART-838 (50 mg/kg/d) or AS (200 mg/kg/d), with three weeks off-treatment between cycles, and 2) four 5d cycles of ART-838 (40 mg/kg/d) or AS (150 mg/kg/d), with one week off-treatment between cycles. Both schedules of ART-838 or AS significantly delayed leukemia onset, confirming results observed using our xenograft model. At these apporoximately equitoxic doses, ART-838 and AS were equally effective against this particular primary B-ALL case. Multiple leukemia cases will need to be tested in this primagraft model in the future to determine if ART-838 has a superior therapeutic window versus AS *in vivo*. Together, these experiments demonstrate the ability of ART-838 or AS to cause substantial *in vivo* inhibition of acute leukemias harboring clinically unfavorable FLT3/ITD or MLLr mutations. Additional cases with different genotypes will be assessed in the future as we determine if further optimization of ART-838 and AS regimens enhance *in vivo* efficacy.

Although in vitro drug combination experiments in cell lines may not fully depict drug interactions in patients, they allow for rational prediction of potentially favorable drug combinations and avoidance of potential antagonism [28]. Combination with ART-838 or AS enhanced the in vitro cytotoxicity of three of the most effective and widely used standard antileukemic drugs (ARA, DOX, and ETO), especially at the high drug concentrations most relevant for clinical cancer treatment [34]. Since artemisinins, as well as ARA, DOX, and ETO, are known to increase intracellular ROS levels [35, 36], one potential mechanism of this drug cooperativity is induction of oxidative stress. Combination of two ROS-inducers might elevate intracellular ROS to levels sufficient to overcome the enhanced endogenous antioxidant capacity of leukemia cells. Further studies are needed to determine the mechanisms of cooperativity of each of these drugs with AS and ART-838, and additional studies should also be performed to specifically test the hypothesis that combination with ART-838 or AS might overcome the development of resistance to antileukemic drugs.

Drugs that overcome resistance to kinase inhibitors are urgently needed to augment the utility of these targeted agents against acute leukemias and other cancers [37–39]. Since several of the most artemisinin-sensitive leukemias we tested harbor FLT3/ITD and/or MLLr mutations, both known to elevate FLT3 signaling, and since multiple other kinases are mutated or overexpressed in acute leukemias, we tested three multi-kinase inhibitors – MID (targeting FLT3, cKit, PDGFR) [40], LES (targeting FLT3, JAK2, TrKA/B/C) [41, 42], and SOR (targeting FLT3, VEGFR, PDGFR, RAF) [43, 44] – in combination with AS or ART-838. SOR has been reported to induce dose-dependent ROS generation in hepatocarcinoma and leukemia cells [45] via targeting of RAF/MEK/ERK pathways [46] and inducing ER stress [47]. Thus, ROS generation may be involved in the additive effects of AS plus SOR. ART-838 combined synergistically with all three kinase inhibitors, possibly because ROS generation may not be the only cytotoxic mechanism of ART-838. Differential interactions of ART-838 versus AS with kinase inhibitors were also recently reported for anti-cytomegalovirus activity [48]. Indeed, these differences in drug combination interactions may aid in elucidating novel mechanisms of action of different artemisinin derivatives.

In summary, ART-838 and AS, members of the widely used, low-toxicity artemisinin class of antimalarial drugs, inhibited all tested human acute leukemia cell lines, representing the three major acute leukemia subtypes, at low micromolar (AS) or submicromolar (ART-838) concentrations. Induction of oxidative stress is an emerging therapeutic strategy for cancers including leukemias, and the pro-apoptotic activity of both ART-838 and AS is at least partially ROS-mediated. PK studies in mice indicated an extended half-life for ART-838, as compared to AS. ART-838 and AS inhibited *in vivo* growth of a leukemia cell line and a primary human acute leukemia case. Based on these in vitro and *in vivo* results, we believe ART-838 and AS worthy of further preclinical development as anti-leukemic agents. Although not fully elucidated, AS targets appear to differ from those of most current cancer chemotherapeutic agents [49], and resistance to AS is not conferred by mechanisms affecting several current antineoplastics [50]. ART-838 might act via additional mechanism(s) as well. ART-838 and AS were found to enhance the antileukemic activity of several established and emerging antileukemic drugs, and further preclinical drug combination studies appear warranted. In addition, since the clinical pharmacology and safety of AS have been established worldwide in malaria treatment, we suggest a Phase I/II clinical dose escalation trial of repeated intermittent 5-10d pulses of oral AS in acute leukemia to find its MTD and further assess PK/PD parameters in humans with leukemia. The results of such a clinical trial would open the door to testing in humans of whether AS enhances clinical efficacy of established and emerging antileukemic drugs, such as SOR and other kinase inhibitors, and/or inhibits the development of drug resistance. If these studies are encouraging, ART-838, with its higher potency and longer *in vivo* half-life as compared to AS, could be further developed with preclinical pharmacologic and animal toxicity studies.

## MATERIALS AND METHODS

### Reagents

Synthesis of ART-838, its parent alcohol ART-606, deoxy-ART-838, and DHA were described [11, 21, 22]. AS was from LKT Laboratories (St. Paul, MN); ETO, ARA, DOX, DFO and NAC from Sigma-Aldrich (St. Louis, MO); LES, MID and z-VAD from Santa Cruz Biotechnology (Dallas, TX); and SOR from LC laboratories (Woburn, MA). ART-838, AS, ETO, LES, MID, SOR, and z-VAD were stored in DMSO (Mediatech, Manassas, VA); ARA and DFO in sterile water; DOX in PBS. All drug stocks were stored at −20°C. NAC was dissolved in culture medium, pH adjusted to 7.4, and filtered immediately before use. Final DMSO concentrations were the same for all samples in any given experiment and always <0.8%.

### Cells

MOLM14 human AML cells (harboring MLLr and FLT3/ITD mutations) were derived from an AML primary sample and provided by Dr. Feyruz Rassool (University of Maryland School of Medicine [UMSOM]) [51]. All other cell lines (Supplementary Table S1) were obtained from American Type Culture Collection (ATCC) or Deutsche Sammlung von Mikroorganismen und Zellkulturen (DSMZ). Cell lines were passaged for <6 months after resuscitation from original cryopreserved stocks. Cell lines were cultured (37°C, 5% CO_2_) in RPMI-1640 containing L-glutamine (Mediatech) and 10% FBS (Gemini Bio-products, West Sacramento, CA). The primary patient B-ALL case (harboring MLLr; uniquely designated B-ALL case #109), was obtained from the Johns Hopkins University School of Medicine cell bank under an Institutional Review Board-approved research protocol). B-ALL #109 cells were expanded and harvested from spleens of transplanted immunodeficient NSG mice [52].

Cryopreserved human CD34^+^ HSPCs from normal adult donors (Cellular Therapy and Cell Processing Facility, Fred Hutchinson Cancer Center, Seattle, WA) were thawed and cultured for 24h in StemSpanSFEM medium (Stemcell Technologies, Vancouver, BC, Canada) containing 100 ng/ml recombinant human (rh) stem cell factor, 20 ng/ml rh thrombopoietin, and 100 ng/ml rh FLT3 ligand (PeproTech, Rocky Hill, NJ) [53], in the presence of the indicated drug concentrations prior to plating for clonogenic assays.

### Cytotoxicity

In most experiments, cytotoxicity was assessed using alamarBlue assays (Life Technologies, Grand Island, NY). In experiments using NAC and DFO, which can interfere with alamarBlue assay performance, Trypan Blue dye exclusion was used instead.

### Clonogenicity

Cells pretreated with ART-838, AS, or vehicle for 24h were diluted and plated in MethoCult medium (Stemcell Technologies). After 7-10d (leukemia cells) or 10-14d (HSPCs), colonies (≥20 cells) were enumerated [53].

### Cell cycle

Cells were pulse-labeled with 10 μM 5-bromo-2-deoxyuridine (BrdU, BD Pharmingen, San Diego, CA) for 90min (37°C), washed in PBS, then fixed in 70% ethanol (1-3d, −20°C). DNA was denatured (2 M HCl, 30min, room temperature) and neutralized (0.1 M Na_2_B_4_O_7_, 10min, room temperature). Cells were stained with APC-conjugated anti-BrdU antibody (BD Pharmingen) and 7-AAD (BioLegend, San Diego, CA), and analyzed by flow cytometry [54].

### Apoptosis

Cells were co-stained with 5 ml each 7-AAD and APC-conjugated Annexin-V (BioLegend), in Annexin-V binding buffer and analyzed by flow cytometry [55]. Caspase-3/7 activity was measured using a Caspase-Glo 3/7 Assay (Promega, Madison, WI).

### ROS

Cells were preloaded with 5 μM 5-(and-6)-chloromethyl-2′,7′-dichlorodihydrofluorescein diacetate (CM-H_2_DCFDA, Life Technologies) in PBS (30min, 37°C), then washed before drug treatment. Following 16h incubation with drugs, cells were analyzed by flow cytometry.

### Mice

Highly immunodeficient NSG mice, obtained originally from Jackson Laboratory (Bar Harbor, ME), were bred and housed in the UMSOM animal facilities, and handled in a laminar flow hood under aseptic conditions. All experiment procedures and animal care were in compliance with the NIH guidelines for the Care and Use of Laboratory Animals and approved by the UMSOM Institutional Animal Care and Use Committee. Drugs were formulated in Tween 80:ethanol (7:3, v:v) and diluted 1:10 in filtered deionized water immediately before administration. For the human AML xenograft model, 6-12-week-old male NSG mice (>20 g) were transplanted sc with MOLM14 cells on day 0 and drug-treated via oral gavage, as specified in legends. Mice were monitored daily and euthanized when tumors reached an estimated mass of 2000mg (using the formula: [length x width^2^]/2, assuming density = 1 mg/mm^3^) [56]. For the human leukemia primagraft model, mice were iv transplanted with primary B-ALL case #109 cells via lateral tail vein on day 0 before drug treatment via oral gavage, as detailed in legends. Mice were euthanized for clinical signs of severe leukemia or other severe distress [52].

### PKs, CBCs

Blood samples were collected via retro-orbital bleeding into EDTA-coated tubes. PK samples were centrifuged to obtain plasma, which was stored frozen (−80°C) until liquid chromatography/triple quadrupole mass spectrometry (LC/MS-MS) analysis. PK parameters were calculated from mean drug concentration-time data using non-compartmental methods as analyzed in Phoenix WinNonlin version 6.3 (Pharsight Corporation, Mountain View, CA) (see Supplementary Table S4 for details) [57]. CBCs were determined using a Hemavet 950 analyzer (Drew Scientific, Oxford CT).

### Statistics

Differences between groups were compared using the Student's *t* test. Prism software (Graphpad, La Jolla, CA) was used for correlation analysis.

## SUPPLEMENTARY FIGURES AND TABLES


