# Supplementary material for: Artemisinin-derived dimer ART-838 potently inhibited human acute leukemias, persisted in vivo, and synergized with antileukemic drugs
Source: Oncotarget. 2016 Jan 12;7(6):7268–79. doi: 10.18632/oncotarget.6896 (PMC4872784; doi:10.18632/oncotarget.6896)
Supplement: Supplementary file 1 [file oncotarget-07-7268-s001.pdf]

SUPPLEMENTARY FIGURES AND TABLES

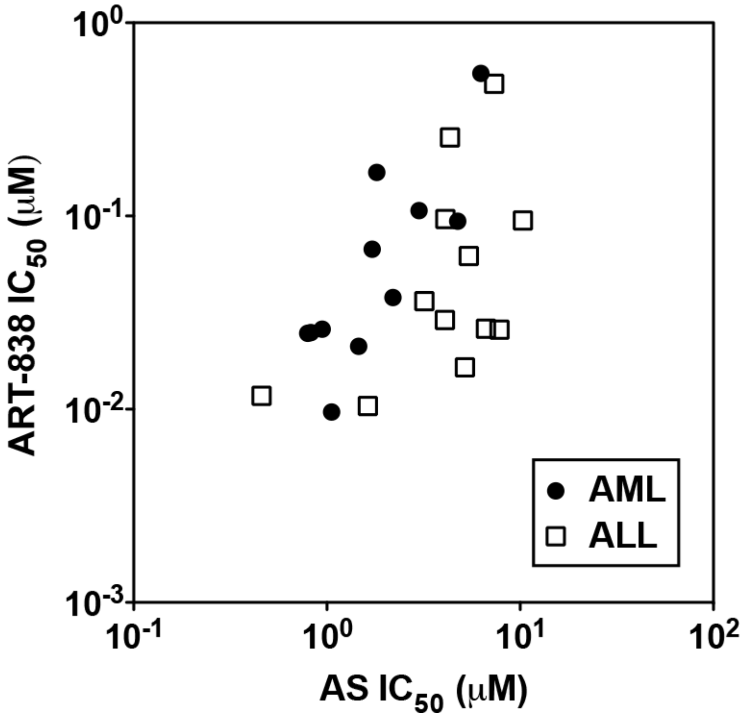

Supplementary Figure S1: Correlation between ART-838 and AS IC_{50}s in 23 human acute leukemia cell lines. ART-838 and AS IC_{50}s from Table S1. Spearman correlation coefficient (r) = 0.5375, $P < .01$.

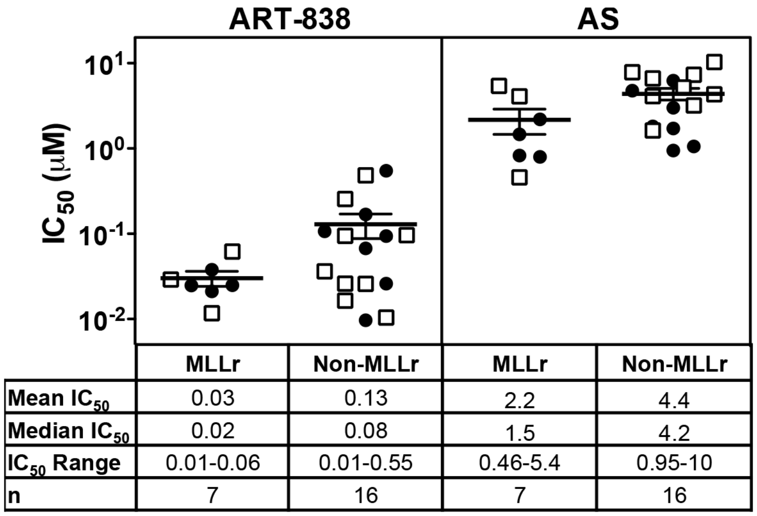

	MLLr	Non-MLLr	MLLr	Non-MLLr
Mean IC_{50}	0.03	0.13	2.2	4.4
Median IC_{50}	0.02	0.08	1.5	4.2
IC_{50} Range	0.01-0.06	0.01-0.55	0.46-5.4	0.95-10
n	7	16	7	16

Supplementary Figure S2: ART-838 and AS IC_{50}s in cell lines with vs without MLLr. IC_{50}s for 7 cell lines with MLLr and 16 cell lines without MLLr from Table S1 (closed circles – AML cell lines, open squares – ALL cell lines). Data are represented as mean +/– SD.

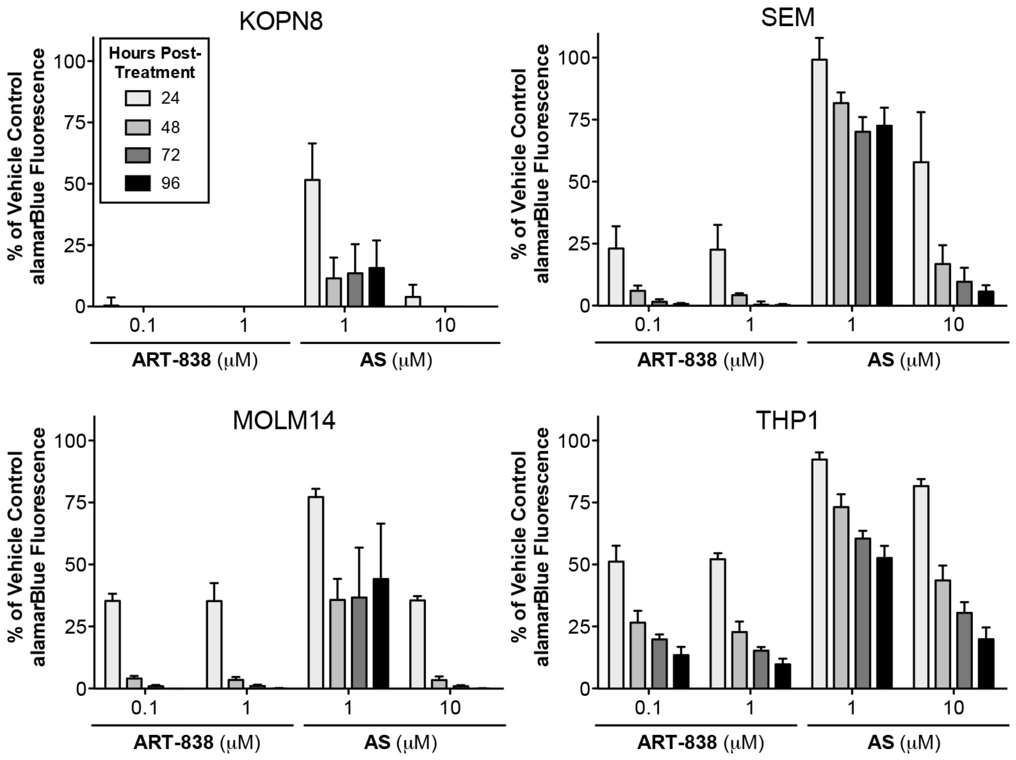

Supplementary Figure S3: Progressive growth inhibition of ALL and AML cell lines by ART-838 and AS over 96 hours. Two B-ALL (KOPN8, SEM) and two AML (MOLM14, THP-1) cell lines were treated with the indicated concentrations of ART-838 or AS for 24–96 h, then viability determined as in Figure 1A. Data are represented as mean +/– SD.

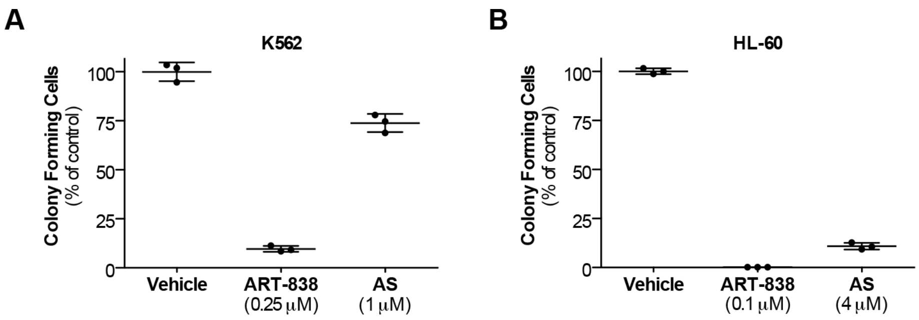

Supplementary Figure S4: ART-838 inhibited clonogenicity of K562 and HL-60 cells. Colony-forming assays were performed as in Figure 1B in **A.** K562 and **B.** HL-60 AML cell lines. Each experiment was performed once in triplicate. Data are represented as mean +/– SD.

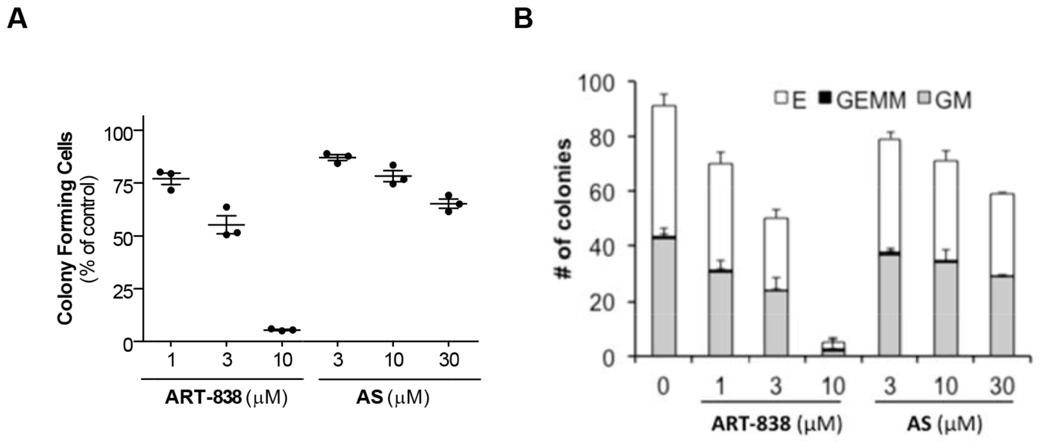

Supplementary Figure S5: Effects of ART-838 and AS on clonogenicity of normal human CD34+ HSPCs. Normal human CD34+ HSPCs were pretreated with the indicated concentrations of ART-838, AS, or vehicle (0.5% DMSO) for 24 h then cultured in semi-solid medium for 10–14d before colonies were enumerated. **A.** Total hematopoietic colony counts, normalized to vehicle-treated controls (100%) and **B.** counts of types of colonies, i.e. erythroid (E); granulocyte/macrophage (sum of GM, G and M, combined) and multilineage progenitors (GEMM). Three independent experiments were performed. Data are represented as mean +/– SEM.

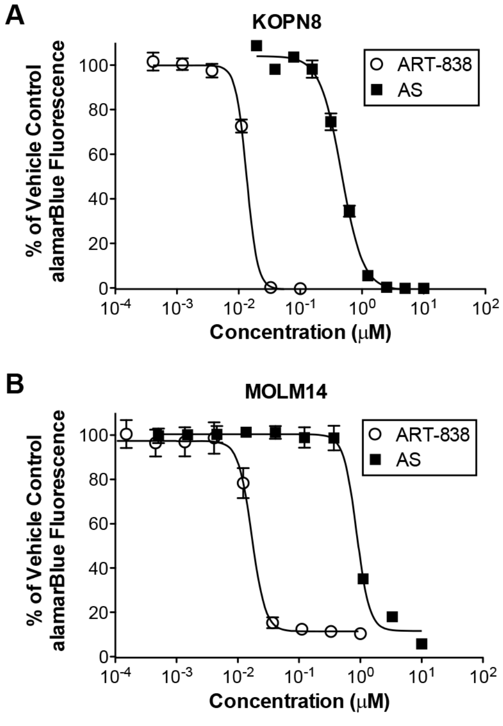

Supplementary Figure S6: ART-838 and AS concentration-response curves for KOPN8 and MOLM14. Concentration-response curves for one representative alamarBlue cytotoxicity assay (in triplicate) used to determine ART-838 and AS IC_{50}s in **A.** KOPN8 and **B.** MOLM14 cells. Data are represented as mean +/– SD.

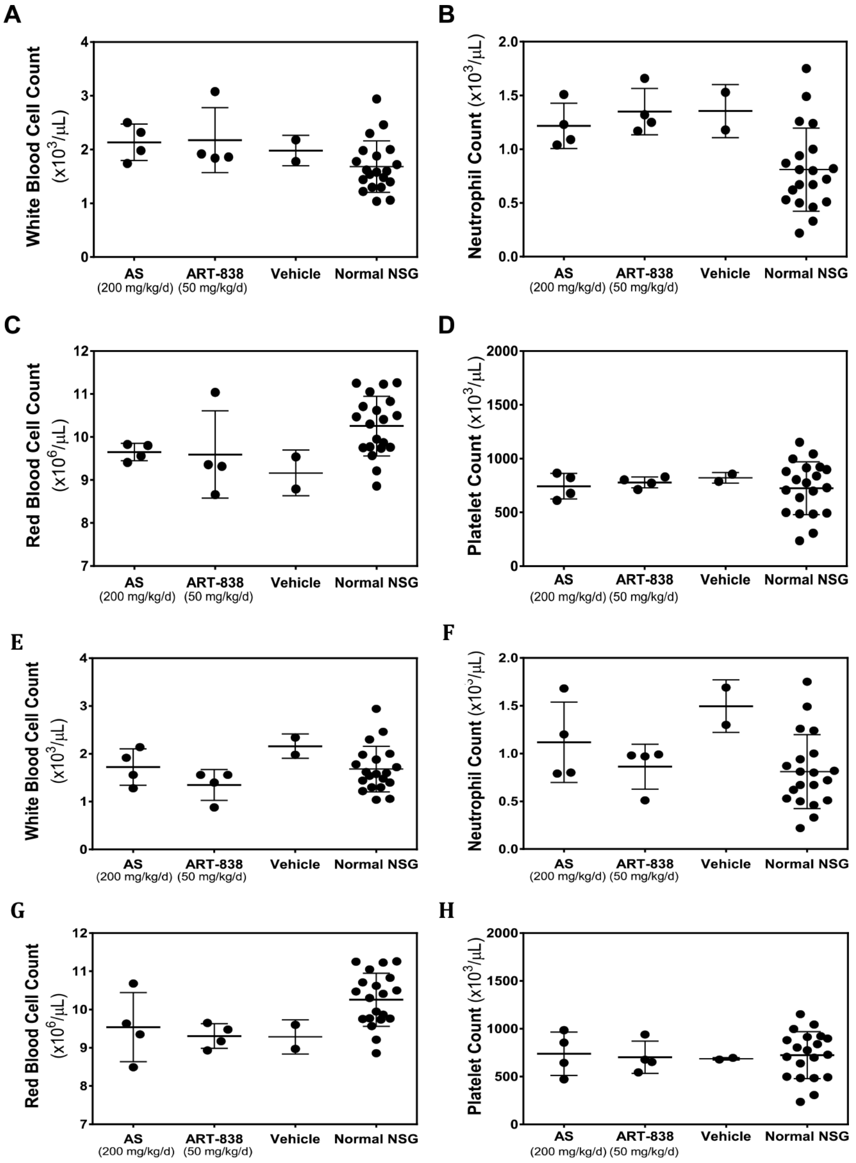

Supplementary Figure S7: Effects of ART-838 and AS on CBCs in normal NSG mice. Blood samples were collected via retro-orbital bleeding into EDTA-coated tubes from MOLM14 NSG mice 1 day **A-D.** and 7 days **E-H.** after completion of a 5-day drug treatment cycle: Vehicle or ART-838 (50 mg/kg/day x 5) or AS (200 mg/kg/day x 5). CBCs were determined using a Hemavet 950 analyzer. Normal blood samples were obtained from normal, untreated NSG mice (*n* = 37) over several years. Data are represented as mean +/– SD.

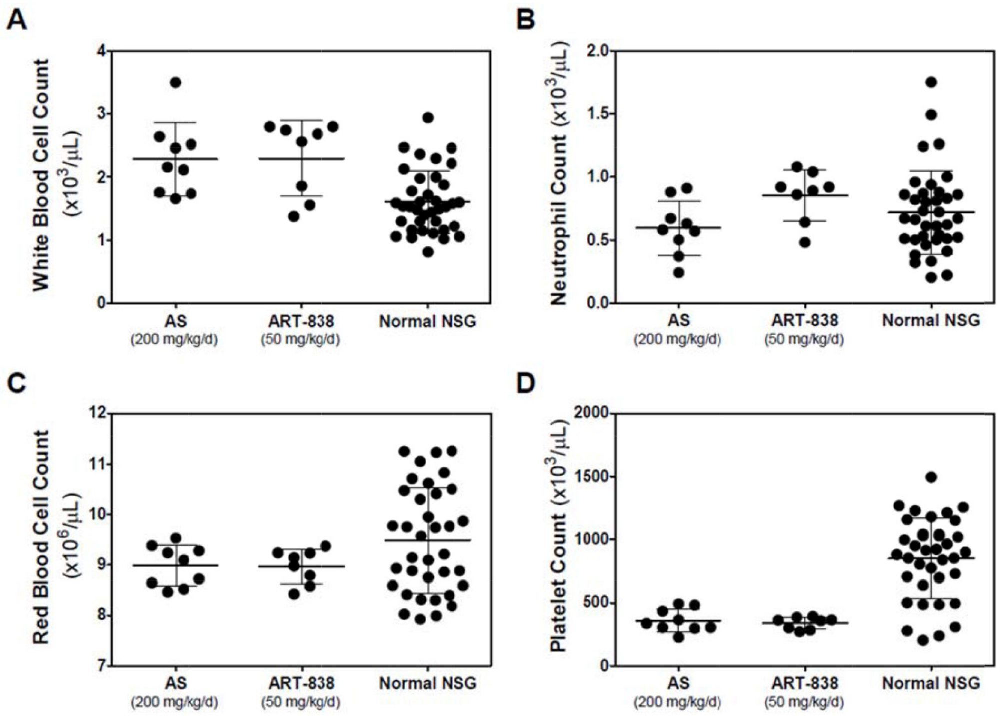

Supplementary Figure S8: Effects of ART-838 and AS on CBCs in tumor-bearing NSG mice. Blood samples were collected via retro-orbital bleeding into EDTA-coated tubes from male MOLM14-tumor-bearing NSG mice 5 days after the completion of the second drug treatment cycle (day 23, Figure 5A) and CBCs determined using a Hemavet 950 analyzer. Normal blood samples were obtained from normal, untreated NSG mice (n = 37) over several years. CBCs were not obtained for vehicle-treated mice, as all had been euthanized by this time point in this experiment. Data are represented as mean +/– SD.

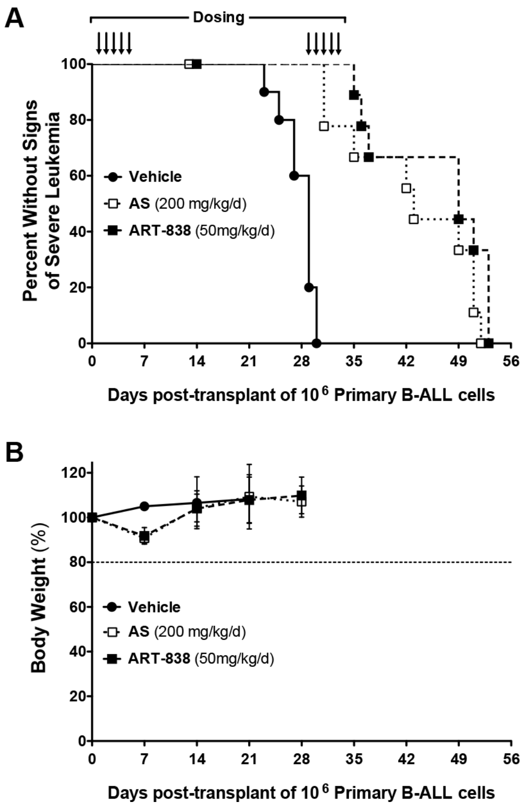

Supplementary Figure S9: ART-838 or AS treatment delayed development of leukemias after transplant of primary human B-ALL cells into NSG mice. NSG mice (*n* = 10 per group) transplanted with primary B-ALL case #109 (10^6 cells/mouse) were treated by oral gavage with two 5-day cycles of ART-838 (50 mg/kg/d), AS (200 mg/kg/d), or vehicle starting on days 1 and 29 post-transplantation (arrows indicate days of drug administration). Mice were weighed weekly and observed daily for clinical signs of severe leukemia. **A.** Kaplan-Meier survival analysis. Mean time to severe clinical signs of leukemia (surrogate for survival): vehicle (28), AS (43), and ART-838 (47); ($p < .001$ for ART-838 vs vehicle or AS vs vehicle). 2 mice (1 each from the AS and ART-838 groups that died on days 13 and 14, respectively) were censored from analysis, as they showed no evidence of leukemia or drug toxicity at necropsy. **B.** Percent of Day 0 body weight. Data in (B) are represented as mean +/– SD.

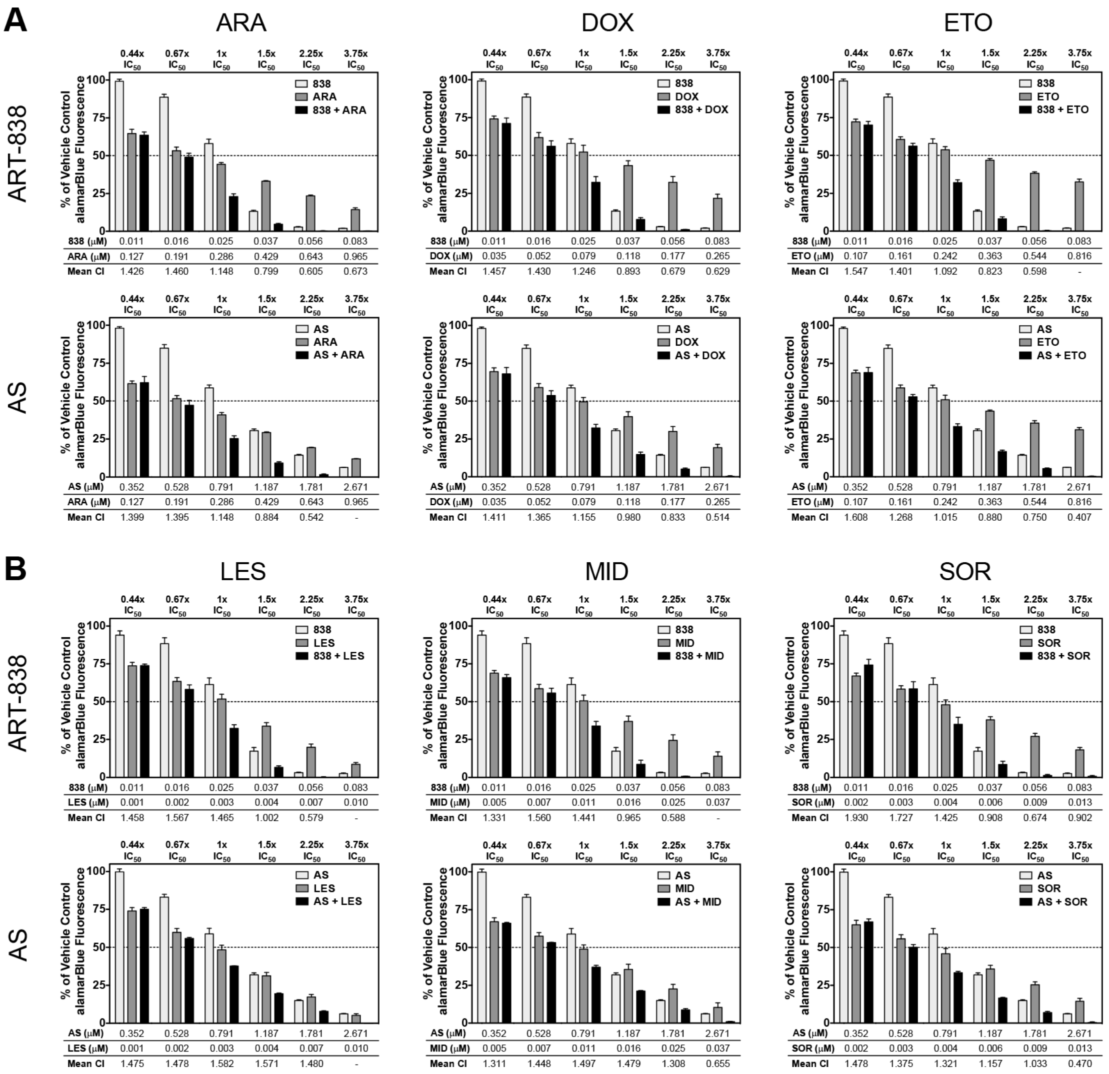

Supplementary Figure S10: Drug combinations against MOLM14. MOLM14 cells were treated with a range of concentrations of ART-838 or AS, alone or in combination with a range of concentrations of standard antileukemic drugs (ARA, DOX, or ETO) or kinase inhibitors (LES, MID, or SOR) at a ratio of the IC_{50}s for each drug for 48 h, and cytotoxicity was assayed using alamarBlue. Each experiment was performed three times in triplicate, and error bars represent the SEM. All panels have error bars but some are too small to be visible. Combination indices (CI) were computed for each drug combination using CompuSyn software, based on the Median Effect Principle and the CI theorem of Chou-Talalay, wherein C*I* = 1 indicates additivity, CI < 1 synergism, and CI > 1 antagonism. Data are represented as mean +/– SD.

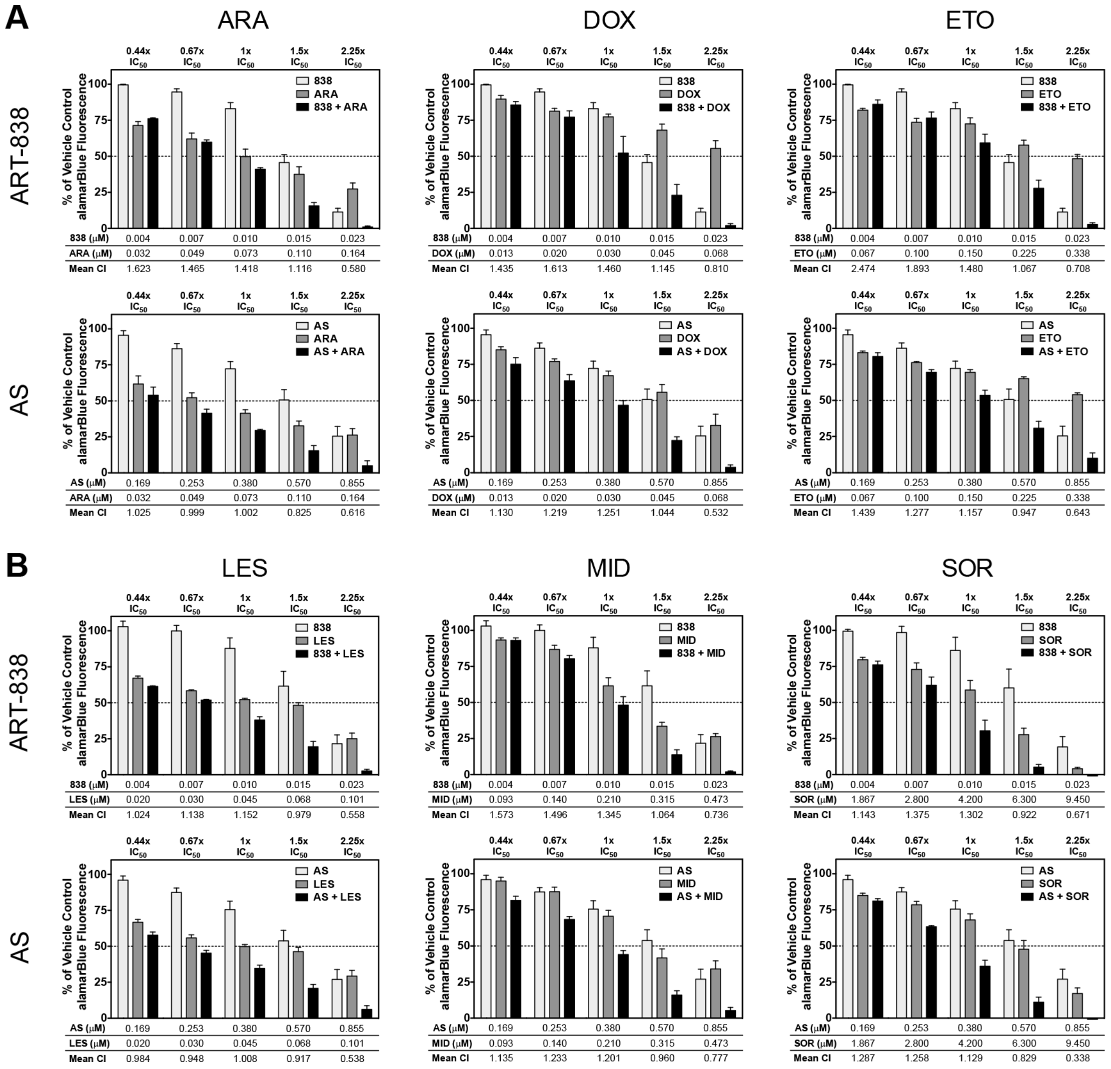

Supplementary Figure S11: Drug combinations against KOPN8. KOPN8 cells were treated with a range of concentrations of ART-838 or AS, alone or in combination with a range of concentrations of **A.** standard antileukemic drugs (ARA, DOX, or ETO), or **B.** kinase inhibitors (LES, MID, or SOR) at a ratio of the IC_{50}s for each drug for 48 h, and cytotoxicity was assayed using alamarBlue. Each experiment was performed at least three times in triplicate, and error bars represent the SEM. All panels have error bars but some are too small to be visible. Combination indices (CI) were computed for each drug combination using CompuSyn software, as above. Data are represented as mean +/– SD.

Supplementary Table S1: ART-838 potently inhibited growth of acute leukemia cell lines *in vitro*

Cell Line	Leukemia Type	Sex	Age (y)	Gene-gene fusions(MLL Fusions in bold)	p53 status	ART-838 IC_{50} (µM)	AS IC_{50} (µM)
KOPN8	B-ALL	F	< 1	**MLL-ENL**	Mut (SM)	0.012±0.002	0.459±0.049
U937	AML	M	37	PICALM-AF10, YY1-WDR25, RPS10-KIAA0090, NCAPG2-ESYT2, HAT1-TRMT11, BSG-TMEM8A	Mut (Fs)	0.010±0.001	1.06±0.17
MV-4–11*	AML/B-ALL**	M	10	**MLL-AF4**	WT	0.025±0.005	0.796±0.096
MOLT-16	T-ALL	F	5	TCRa/c-MYC, ELAVL1-TYK2	Mut (DM)	0.010±0.002	1.63±0.07
MOLM14*	AML	M	20	**MLL-AF9**	–	0.025±0.003	0.825±0.082
ML-2	AML	M	26	**MLL-AF6,** HECTD1-STK4, ARID4A-NCOA5	Mut (SM)	0.021±0.011	1.46±0.21
NB-4	AML	F	23	PML-RARA	WT	0.026±0.007	0.947±0.141
NALM-6	B-ALL	M	19	TEL-PDGFRβ	WT	0.016±0.005	5.19±0.43
THP-1	AML	M	1	**MLL-AF9**	Mut (Fs)	0.038±0.009	2.20±0.48
Kasumi-1	AML	M	7	AML1-ETO, RUNX1-RUNX1T1, PROSC-WHSC1L1, MPO-RPLP1	Mut (SM)	0.067±0.023	1.72±0.23
SEM	B-ALL	F	5	**MLL-AF4**	–	0.029±0.006	4.08±1.18
RCH-ACV	B-ALL	F	8	E2A-PBX1	WT	0.036±0.012	3.20±0.54
OCI-AML3	AML	M	57	USP34-CCT4, SREBF2-MEI1	WT	0.168±0.041	1.81±0.20
SUPB15	B-ALL	M	8	BCR-ABL1, UBE2K-PDS5A,	WT	0.026±0.003	7.82±1.22
Kasumi-2	B-ALL	M	15	E2A-PBX1	–	0.026±0.004	6.65±0.34
HL-60	AML	F	36	YTHDC1-TXNL4A, NOC4L-FBRSL1, EDF1-C9orf86, CYFIP2-PLCG2	Null	0.107±0.045	3.00±0.60
RS4;11	B-ALL	F	32	**MLL-AF4**	WT	0.062±0.023	5.42±1.89
K562	CML**	F	53	BCR-ABL1, RPS18-MAK16, RPS20-IARS	Mut (Fs)	0.094±0.012	4.75±0.85
MOLT-3	T-ALL	M	19	–	–	0.097±0.058	4.10±0.62

(*Continued*)

Cell Line	Leukemia Type	Sex	Age (y)	Gene-gene fusions(MLL Fusions in bold)	p53 status	ART-838 IC_{50} (µM)	AS IC_{50} (µM)
Jurkat	T-ALL	M	14	YTHDC2-ST13, WDSUB1-NUF2, TSSC1-TSPAN7, SLC25A12-C11orf46, RPL12-RGCC, LEF1-HADH, GRAMD4-SET, FAM1338-CDK6, AZI1-ACAP1	Mut (MM)	0.255±0.155	4.33±1.18
REH	B-ALL	F	15	TEL-AML1	WT	0.095±0.040	10.3±3.0
KG-1a	AML	M	59	FGFR1OP2-FGFR1, PPP1R9A-XRCC2	Mut (SM)	0.547±0.147	6.26±0.30
CCRF-CEM	T-ALL	F	3	–	Mut (MM)	0.483±0.195	7.34±1.24

IC_{50}s (plotted in Figure 1A) were calculated using CompuSyn software and represent the means of at least three experiments performed in triplicate. Cell lines are ordered by average rank sensitivity to ART-838 and AS. Age refers to age at the time of leukemia diagnosis of the patient from whom the cell line was derived. (–) Indicates information unavailable.
[*]Cell lines with FLT3/ITD mutations.
[*]The MV-4–11 cell line was derived from a patient with a biphenotypic B-myelomonocytic leukemia; K562 was derived from a patient with chronic myeloid leukemia in myeloid blast crisis; both are classified as AMLs for the purposes of Figure 1A. p53 status was obtained from http://cancer.sanger.ac.uk/cell_lines/cbrowse/all and http://p53.free.fr/Database/Cancer_cell_lines/Leukemia_cancer. html. WT: wild type. Mut: mutant. SM: single mutation. DM: double mutation. MM: multiple mutations. Fs: Frameshift mutation. Null: protein expression undetectable. Data are represented as mean +/– SD.

Supplementary Table S2: ART-838 and AS IC_{50}s differ depending on culture media

Cell Line	Original Medium	ART-838 IC_{50} in original medium, µM	ART-838 IC_{50} in RPMI+10% FBS, µM	-Fold Difference in ART-838 IC_{50} between media	AS IC_{50} in original medium, µM	AS IC_{50} in RPMI+10% FBS, µM	-Fold Difference in AS IC_{50} between media
SEM	DMEM + 10% FBS	0.268	0.029	9.2	18.30	4.08	4.5
THP-1	RPMI + 10% FBS + 50 µM β-mercaptoethanol	0.095	0.038	2.5	11.07	2.20	5.0
OCI-AML3	RPMI + 20% FBS	0.287	0.168	1.7	3.56	1.81	2.0
SUPB15	RPMI + 20% FBS	0.077	0.026	3.0	14.34	7.82	1.8
REH	RPMI + 20% FBS	0.123	0.095	1.3	15.44	10.33	1.5

5 cell lines were initially cultured and tested for AS- and ART-838-induced cytotoxicity via alamarBlue assay in "original media" other than RPMI supplemented with 10% FBS, then were transitioned to this standard culture medium and IC_{50} determinations repeated, yielding lower IC_{50}s for all 5 lines. Therefore, we caution that the culture medium utilized may influence observed IC_{50}s for artemisinins.

Supplementary Table S3: IC_{50}s for DHA, deoxy-ART-838, and 6 antileukemic drugs in KOPN8 and MOLM14 cells

Cell Line	deoxy-ART-838 IC_{50}, µM	ARA IC_{50}, µM	DOX IC_{50}, µM	ETO IC_{50}, µM	MID IC_{50}, µM	LES IC_{50}, µM	SOR IC_{50}, µM	DHA IC_{50}, µM
KOPN8	30.5	0.073	0.030	0.150	0.210	0.045	4.20	0.351
MOLM14	48.5	0.286	0.079	0.242	0.011	0.003	0.004	1.02

KOPN8 and MOLM14 cell lines were treated with a range of concentrations of each drug for 48 h, then cytotoxicity was measured via alamarBlue assay. Results were normalized to cells treated with vehicle (≤0.5% DMSO) alone. IC_{50}s were calculated using CompuSyn software and represent the means of at least three experiments performed in triplicate.

Supplementary Table S4: Pharmacokinetics of ART-838, AS, and DHA in NSG mice

Drug	$T_{1/2}$, h	T_{max}, h	C_{max}, ng/ml	AUC_{last}, h*ng/ml	$AUC_{0-\infty}$, h*ng/ml	Vz/F, L/kg	CL/F/kg, L/h/kg
ART-838	3.19	1.5	383	903	951	241	52
AS	NR	0.25	67	34.8	NR	NR	NR
DHA	0.58	0.25	1629	1293	1305	103	123

ART-838 or AS and known/potential metabolites were extracted from 50 µL plasma using protein precipitation. Analytes were separated using an Agilent Zorbax Eclipse C_{18} column (2.1 mm x 50 mm, 3.5 µm) using gradient mobile phase (acetonitrile and 2 mM ammonium acetate both containing 0.1% formic acid) and a gradient flow rate (0.2 to 0.4 ml/min). An AB Sciex 5500 triple quadrupole mass spectrometer was used for detection. The m/z transitions used were 407.0→261.1 (AS), 302.2→163.1 (DHA [known metabolite of AS]), 856.33→589.30 (ART-838), 629.03→537.3 (ART-606 [possible metabolite of ART-838]), and 300.1→209.2 (artemisinin [internal standard]). The calibration range was 5–1,000 ng/mL for all analytes. Mean drug concentrations were calculated at each time point. PK parameters (plasma half-life [$T_{1/2}$]; time to reach C_{max} [T_{max}]; maximum plasma concentration [C_{max}]; area under the plasma concentration-time curve [AUC] from time zero to the last measurable concentration [AUC_{last}]; AUC from time zero to infinity [i.e., the AUC_{last} + the extrapolated AUC from AUC_{last} to infinity; $AUC_{0-\infty}$]; apparent volume of distribution [Vz/F]; body weight-adjusted apparent clearance [CL/F/kg]) were calculated from mean drug concentration-time data using non-compartmental methods as analyzed in Phoenix WinNonlin version 6.3. If the percent AUC extrapolated was > 25% or the r^2 of the elimination rate constant (λ_z) was < 0.9, the $AUC_{0-\infty}$, CL/F, and $T_{1/2}$ were not reported (NR).
